# Cerebral Autosomal Dominant Arteriopathy With Subcortical Infarcts and Leukoencephalopathy (CADASIL): A Diagnosis to Consider in Atypical Stroke Presentations

**DOI:** 10.7759/cureus.46482

**Published:** 2023-10-04

**Authors:** Jing W Goh, Satyaki Kundu, Ragunath Durairajan

**Affiliations:** 1 Acute Medicine, Russells Hall Hospital, Dudley, GBR; 2 Internal Medicine, Russells Hall Hospital, Dudley, GBR

**Keywords:** subcortical ischemic leukoencephalopathy (cadasil), notch 3, stroke, cadasil, cerebral autosomal dominant arteriopathy with subcortical infarcts and leukoencephalopathy (cadasil)

## Abstract

CADASIL (cerebral autosomal dominant arteriopathy with subcortical infarcts and leukoencephalopathy) is a hereditary cerebral arteriopathy caused by a neurogenic locus notch homolog protein 3 (NOTCH3) gene mutation. This article describes the case of a man in his early 40s who experienced sudden onset temporary left-sided weakness and facial numbness that resolved in two hours, along with residual weakness in the upper and lower limbs. There was a family history of CADASIL with both his mother and brother having experienced strokes. Clinical investigations on admission were unremarkable including a young stroke screen and computed tomography (CT) of the brain. On CT angiography, there was no evidence of significant carotid artery stenosis. Subsequent cerebral magnetic resonance imaging (MRI) revealed an acute infarction in the right corona radiata extending into the right basal ganglia, with the fluid-attenuated inversion recovery (FLAIR) images revealing considerable bilateral symmetrical white matter hyperintensity in the frontal, parietal, and anterior temporal lobes. Based on his clinical presentation, strong family history, and brain MRI findings, a diagnosis of CADASIL was suspected. He received antiplatelet medication for an acute stroke and later underwent genetic testing, which revealed the presence of a NOTCH3 gene mutation.

## Introduction

CADASIL, or cerebral autosomal dominant arteriopathy with subcortical infarcts and leukoencephalopathy, is a rare hereditary cerebral arteriopathy caused by mutations in the notch homolog protein 3 (NOTCH3) gene on chromosome 19p13, which makes a transmembrane receptor protein whose function is unknown. The pathogenic NOTCH3 receptor protein builds up in the small and medium-sized cerebral arteries, causing cerebral infarctions brought on by fibrosis and arterial wall thickening, resulting in vascular smooth muscle cell degeneration and multiple small infarcts in the brain's white and deep gray matter [1].

CADASIL is the most common hereditary stroke syndrome, manifesting in early adulthood as an acute and progressive stroke with subcortical vascular dementia [2]. However, because it is a rare clinical phenomenon, there has been little research on its prevalence and incidence. According to Razvi et al.'s 2005 study, it affects 1.98 out of every 100,000 people in West Scotland [3]. CADASIL can be difficult to diagnose on the initial presentation due to its generic clinical characteristics, with a large proportion of confirmed CADASIL initially presenting as migraine headaches and even mood disorders. Furthermore, risk factors for vascular stroke are not always present in CADASIL. Brain MRI abnormalities such as anterior temporal lobe involvement are generally specific in the early stages of CADASIL and can provide good diagnostic clues to CADASIL [4]. However, investigations have shown that anterior temporal lobe involvement is not pathognomonic and can be detected in people who do not have the NOTCH3 genetic mutation [5]. As a result, the diagnosis of CADASIL mainly relies on the examination of the NOTCH3 genetic sequence, which has a sensitivity of over 95% for detecting NOTCH3 genetic abnormalities [6]. More than 90% of CADASIL patients are expected to have a defective NOTCH3 gene [7].

## Case presentation

A 41-year-old male presented to the emergency department (ED) with a rapid onset of left-sided facial numbness that progressed to his left arm and leg, as well as left-sided weakness in his upper and lower limbs coupled with ataxia while at work. He had no visual or speech disturbances. The symptoms lasted for two hours and progressively subsided, leaving him with a mild headache and ongoing weakness in his left arm and leg 30 minutes after arriving at the ED.

He was otherwise in good health with no significant past medical history including migraines or other vascular conditions. He had no history of headaches, mood disturbance, or symptoms of neurological impairment. His mother, who was 55, and his brother, who was 38, were both diagnosed with CADASIL. He did not receive genetic screening for CADASIL prior to this presentation. He worked as a metalworker and occasionally consumed alcohol on weekends. He has been smoking cigarettes for the past 25 years and previously smoked cannabis and there was no history of illicit drug use. There was an absence of any respiratory, cardiovascular, abdominal, and urinary symptoms.

On presentation, his vital signs were normal, and the National Institute of Health Stroke Scale (NIHSS) score was 2. Clinical examination identified weakness in his left upper and lower limbs, with a muscle power of four on the Medical Research Council's (MRC) scale. Cranial nerves II-XII were intact. The rest of the physical examination was unremarkable. He was then admitted to the stroke ward for further investigations and management.

Investigations

**Figure 1:**
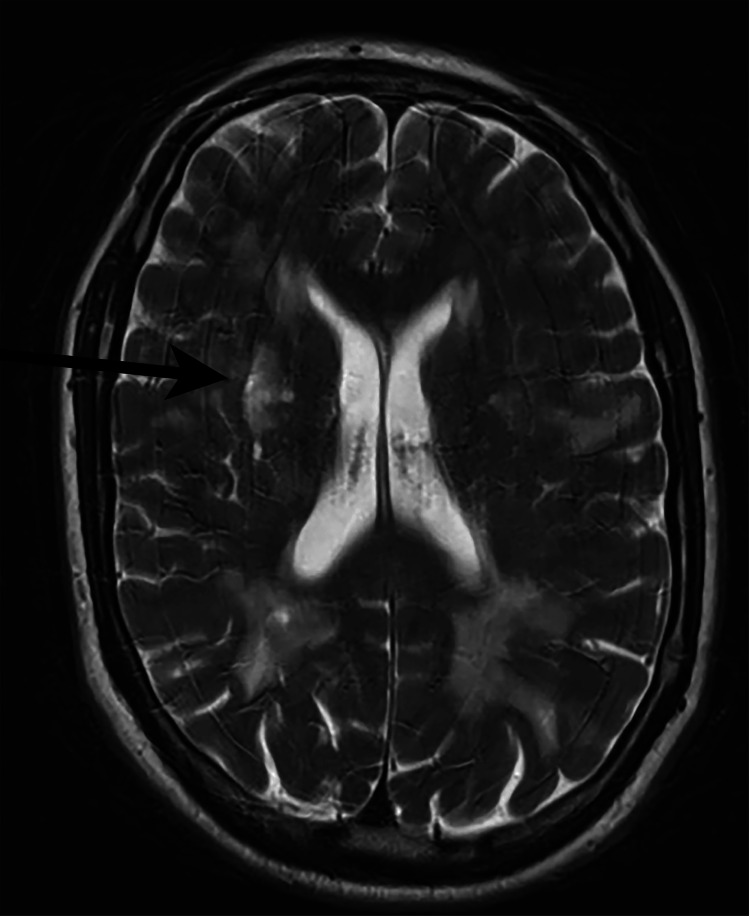
MRI head: Black arrow showing acute infarct in the right corona radiata MRI: magnetic resonance imaging

**Figure 2:**
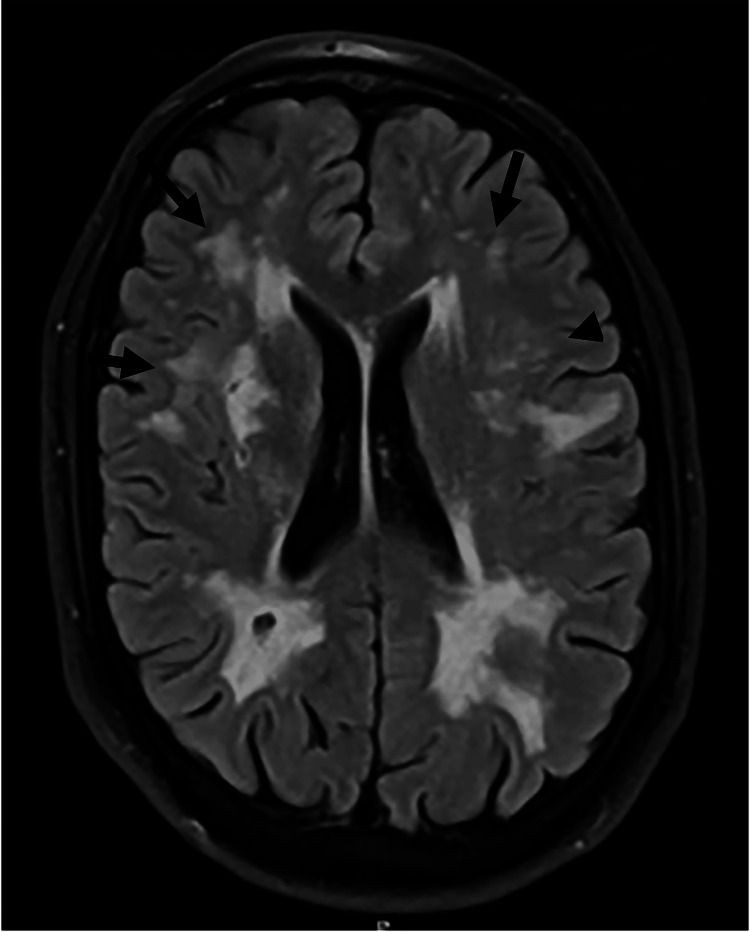
MRI head T2: Black arrows showing white matter hyperintensities in the frontal, parietal lobes extending into the anterior temporal lobes MRI: magnetic resonance imaging

He had undergone a series of imaging and blood investigations to rule out other causes of stroke. The initial blood screening for young stroke patients found no significant abnormalities (see Table 1). His CT brain showed ill-defined low attenuation in the right lentiform nucleus, which raised the clinical suspicion of acute ischemia. As a result, an MRI was performed, which revealed an acute infarct in the right corona radiata that extended into the right basal ganglia (see Figure 1). Fluid-attenuated inversion recovery (FLAIR) imaging revealed symmetrical bilateral white matter hyperintensities in the frontal, parietal, and anterior temporal lobes (see Figure 2). There was no proximal large vessel obstruction or carotid stenosis indicated by the CT angiogram. The ECG revealed sinus rhythm with left bundle branch block (LBBB), and echocardiography revealed mild left ventricular systolic dysfunction with an ejection fraction of 49% and dyssynchronous and hypokinetic LV, most likely due to the presence of the LBBB.

**Table 1:** Laboratory results during admission ACE: angiotensin-converting enzyme, TSH: thyroid-stimulating hormone, IgG: immunoglobulin G

Labs (Unit)	Normal Range	Value
Plasma		
Haemoglobin	120-160	153
Mean Cell Volume	78-98 FL	85.6
White Cell Count	4-11 x 10^ 9/L	11.80 ­
Platelets	140-400 x 10^ 9/L	456 ­
Coagulation profile	-	Within Normal Limits
ACE	< 40mcg/L	20
TSH	0.4-4.0mlU/L	1.59
Antinuclear Antibody	-	Negative
Lupus Anticoagulant screen	-	Negative
IgG Cardiolipin Antibody	-	Negative
Syphilis serology	-	Negative
Triglyceride	<2.3mmol/L	5.0 ­­­
HIV antibodies	-	Negative
SARS-CoV-2 RNA	-	Not detected

Outcome and follow-up

On the basis of MRI findings, a clinical picture of stroke, and a significant family history of CADASIL, he was provisionally diagnosed with CADASIL. He was given aspirin and atorvastatin to treat his stroke. On the following day, he was discharged with aspirin 300mg once daily for seven days, followed by clopidogrel 75mg once daily. He was later scheduled for a NOTCH3 genetic sequencing test, which indicated the presence of the family pathogenic variant c.397C>T p.(Arg133Cys). ​​His children and family members were referred to geneticists for genetic counseling.

He could still perform all activities of daily living, although he is currently unemployed due to left-sided residual upper and lower limb weakness. He mobilizes with a stick and can only walk 10 meters on his own. After his hospital discharge, he will be followed up in a stroke outpatient clinic to assess progress.

## Discussion

The case report illustrates the clinical rarity as well as the difficulties in diagnosing CADASIL. Clinical signs of CADASIL differ from person to person and are frequently less specific. Migraine, cognitive dysfunction, recurrent transient ischemic episodes (TIA), and ischaemic stroke are all common in CASADIL patients, with up to 71% experiencing TIAs or strokes and 38% experiencing recurrent headache, the majority of which was linked with auras [8]. Patients typically exhibit the features of cerebral ischemic attacks, which frequently take the form of lacunar stroke [9]. Young individuals may experience migraine attacks at the early stages of CADASIL which may result in complicated migraine symptoms or focal neurologic deficits [10].

Clinical presentation of CADASIL is highly variable and so it is rarely considered as one of the initial differential diagnoses. Recurrent headaches at the early stage of the disease are often being misdiagnosed as migraine. A study by Guey et al. reported that up to 54% of the people had migraine, and 84% of them having migraine with aura prior to the diagnosis of CADASIL [11]. Matar et al. reported a late diagnosis of CADASIL in a young patient who initially presented with recurrent migraine episodes and new onset of hemiplegia, which was previously misdiagnosed as hemiplegic migraine. A brain MRI and a genetic test for NOTCH3 were used to confirm the diagnosis of CADASIL after the patient revealed that her brother had been given an undefined neurological condition [12].

There is a limitation in the present diagnostic criteria for CADASIL, such as that outlined by the International Classification of Headache Disorders (ICHD) 2018 for those presenting with recurrent headaches. One of the criteria is a history of migraine with aura or recurring migraine attacks with aura. Although the majority of CADASIL patients exhibit recurrent headaches with aura, it has been reported that up to 9% of patients present with migraines without aura [13]. As a result, the authors propose a revision in the diagnostic criteria to include migraine without aura.

Diagnosing CADASIL can be challenging due to the variability of its symptoms. The patient mentioned in this case report had no prior history of migraine symptoms or recurrent headaches. Prior to the CADASIL diagnosis, he also showed no symptoms of neurological impairment or psychological issues. As a result, clinicians commonly use MRI to aid with CADASIL diagnosis. It has been reported that MRI with anterior temporal white matter alterations has a high sensitivity of 90% with a specificity of 100% for the diagnosis of CADASIL [4]. The MRI of our patient confirmed the classic CADASIL MRI appearance with an acute infarct in the right corona radiata, right basal ganglia, and symmetrical bilateral involvement of the frontal, parietal, and anterior temporal lobes. The specificity of these MRI findings is however limited because they could also be present in patients lacking NOTCH3 mutations [14]. CADASIL is difficult to distinguish from other forms of cerebral small vascular disease just on the basis of MRI. Therefore, the presence of the aforementioned MRI abnormalities would contribute to the probability of CADASIL rather than being pathognomonic of CADASIL diagnosis.

The gold standard for diagnosing CADASIL continues to be genetic testing for NOTCH3 genetic mutations through genomic sequencing or detection of granular osmiophilic material (GOM) via skin biopsy. These examinations, nevertheless, can be time- and money-consuming, which led to the development of a screening standard for the diagnosis of CADASIL. In 1997, Davous proposed a CADASIL diagnostic standard. Later, in 2017, Mizuta et al. suggested a new diagnostic standard that has a high sensitivity of 97% when screening individuals for CADASIL [15]. However, neither CADASIL nor a family history of its symptoms were included in the suggested criteria. The authors of this case report emphasize the importance of eliciting family history in CADASIL diagnosis in clinical practice. Notably, our patient's strong family history and his early onset of ischemic stroke have contributed to the later investigations for CADASIL. Due to his brother's early TIA presentation, CADASIL was explored. When his mother's stroke symptoms began in her mid-50s, a genetic test for NOTCH3 revealed that she had CADASIL. They did not have neurological impairment, recurrent headaches, or psychosocial issues prior to receiving a CADASIL diagnosis. In order to aid clinicians in identifying CADASIL, the authors of this case report suggest including a family history of symptoms or CADASIL diagnosis in screening.

We conclude by emphasizing that CADASIL syndrome should be suspected in individuals who have TIAs or acute strokes and have no risk factors, family history of stroke, or specific neuroimaging findings. Further testing is warranted in this group with NOTCH3 gene mutation testing.

## Conclusions

CADASIL is a rare hereditary condition that leads to the early onset of stroke. In light of the broad spectrum of its clinical manifestations, it is frequently misinterpreted as a TIA, stroke, or migraine. This case report describes the unusual manifestation of CADASIL in our patient, who did not initially present with any symptoms suggestive of CADASIL. It should be suspected in young individuals presenting with TIA or stroke-like symptoms or recurrent migraine attacks. Other factors such as a positive family history or similar symptoms in first-degree relatives should strengthen the clinical suspicion further. MRI findings are very helpful in aiding diagnosis though they are not specific for this condition. Genetic testing is the gold standard for making the diagnosis.
